# Cow’s Milk-related Symptom Score (CoMiSS) values in presumed healthy European infants aged 6–12 months: a cross-sectional study

**DOI:** 10.1007/s00431-023-05334-0

**Published:** 2023-11-17

**Authors:** Mateusz Jankiewicz, Fatme Ahmed, Katerina Bajerova, Maria Eva Carvajal Roca, Christophe Dupont, Koen Huysentruyt, Mikael Kuitunen, Rosan Meyer, Rouzha Pancheva, Carmen Ribes Koninckx, Silvia Salvatore, Raanan Shamir, Annamaria Staiano, Yvan Vandenplas, Hania Szajewska

**Affiliations:** 1 Department of Paediatrics, The Medical University of Warsaw, Żwirki I Wigury 63A, 02-091 Warsaw, Poland; 2 Department of Hygiene and Epidemiology, Faculty of Public Health, Medical University of Varna, Varna, Bulgaria; 3 Research Group NutriLect, Department of Neuroscience, Research Institute, Medical University “Prof. Dr. Paraskev Stoyanov”, Varna, Bulgaria; 4 Department of Pediatrics, University Hospital Brno and Masaryk’s University, Brno, Czech Republic; 5 Department of Internal Medicine, Geriatrics and Practical Medicine, University Hospital Brno and Masaryk´S University, Brno, Czech Republic; 6 Hospital La Salud, Universidad Católica de Valencia San Vicente Mártir, Valencia, Spain; 7 Ramsay Group, France Et Clinique Marcel Sembat, Paris Descartes University, Boulogne-Billancourt, Paris, France; 8 Vrije Universiteit Brussel (VUB), UZ Brussels, KidZ Health Castle, Laarbeeklaan 101, Brussels, Belgium; 9 Children’s Hospital, University of Helsinki and Helsinki University Hospital, Helsinki, Finland; 10 Department Paediatrics, Imperial College London, London, SW7 2BX UK; 11 Department Dietetics, Winchester University, Winchester, SO23 4NR UK; 12 Department Medicine, KU Leuven, Louvain, Belgium; 13 Pediatric Gastroenterology, La Fe University Hospital, Instituto de Investigation Sanitaria La FE Valencia, Valencia, Spain; 14 Department of Paediatrics, University of Insubria, Varese, Italy; 15 Institute of Gastroenterology, Nutrition and Liver Diseases, Schneider Children’s Medical Center, Lea and Arieh Pickel for Pediatric Research, Faculty of Medicine, Tel-Aviv University, Tel-Aviv, Israel; 16 Department of Translational Medical Science, Section of Pediatrics, University Federico II, Naples, Italy

**Keywords:** Cow’s milk allergy, Awareness, Tool, Infant feeding, CoMiSS

## Abstract

The Cow’s Milk-related Symptom Score (CoMiSS) is an awareness tool for evaluating cow’s milk-related symptoms. Previous studies have focused on providing CoMiSS values for healthy and symptomatic infants aged 0–6 months. However, there is a notable gap in the literature concerning CoMiSS values for infants older than 6 months. This cross-sectional study aimed to determine CoMiSS values in presumed healthy infants who have completed 6 months and are up to 12 months old, hereafter referred to as 6 to 12 months old. Physicians from six European countries prospectively determined CoMiSS values in infants attending well-child clinics. Exclusion criteria included preterm delivery, acute or chronic disease, and the consumption of a therapeutic formula, dietary supplements (except vitamins), or medication. The following information was collected: gestational age, gender, age, type of feed (breast milk or infant formula), and complementary feeding. Descriptive statistics were summarized with mean and standard deviation for normally distributed continuous variables, median and IQR for non-normally distributed variables, and differences in CoMiSS values were analyzed with appropriate tests. Data from 609 infants were obtained. The overall median (Q1–Q3) CoMiSS values were 3 (1–5). Significant differences were found across age groups (*p* < 0.001), but not across groups based on gender (*p* = 0.551) or feeding type (*p* = 0.880).

*   Conclusions*: This study provided CoMiSS values in presumed healthy infants aged 6–12 months. Additional studies should be conducted to establish the use of CoMiSS to assess cow’s milk-related symptoms in infants 6 months and older.

**Table:** 

**What is Known:** *• The Cow’s Milk-related Symptom Score (CoMiSS) is an awareness tool for evaluating symptoms related to cow’s milk.* *• CoMiSS values for presumed healthy infants aged 0-6 months infants are already available.*	
**What is New:** *• CoMiSS values in European infants aged 6-12 months are provided.* *• These CoMiSS values differed across various age groups but not across groups based on gender or feeding type.*

**Supplementary Information:**

The online version contains supplementary material available at 10.1007/s00431-023-05334-0.

## Introduction

Cow’s milk allergy (CMA) is a common food allergy in infants and young children. However, the prevalence varies based on geography, feeding methods, and assessment methods [1]. For instance, a recent systematic review from European countries found that the overall pooled estimates for self-reported lifetime prevalence of cow’s milk allergy is 5.7% [2, 3]. However when verified through food challenges, the prevalence drops to less than 1% [3, 4]. These rates are even lower, ranging from 0.4 to 0.5%, in exclusively breastfed infants [5, 6]. Misdiagnosis is a common issue, and overdiagnosis in some settings is of particular concern [7, 8]. Given the potential long-term health implications [9, 10], a diagnosis of CMA should be made cautiously. The diagnosis of immunoglobulin E (IgE)-mediated allergy is relatively easy because of its immediate symptom onset and the availability of validated tests to confirm the clinical history. However, non-IgE-mediated CMA presents a diagnostic challenge, as its symptoms often overlap with common functional gastrointestinal disorders, such as infantile colic or gastroesophageal reflux [11].

The Cow’s Milk-related Symptom Score (CoMiSS) was developed in 2015 as an awareness tool for healthcare professionals, to evaluate cow’s milk-related symptoms in infants aged 0–6 months [12]. It provides a total score, as well as subscale scores, based on the assessment of crying, regurgitation, stools, skin, and respiratory symptoms evaluated by a healthcare professional in the absence of infectious disease. Crying duration is scored from 0 (less than 1 h) to 6 (more than 5 h per day). The regurgitation score depends on volume and frequency, ranging from 0 (less than 2 episodes per day) to 6 (regurgitation of the complete feed after each feeding). Stool consistency is assessed with the Brussels Infant and Toddlers Stool Scale (BITSS), with scores ranging from 0 (formed stools) to 6 (watery stools). Skin symptoms include atopic eczema severity scores for different body regions for more than 1 week on head, neck, trunk (scores of 0–3) and extremities (scores of 0–3) and acute urticaria and/or angioedema (score of 6). Respiratory symptoms scores range from 0 (no respiratory symptoms) to 3 (severe symptoms). The total CoMiSS score ranges from 0 to 33 (Table 1).

**Table 1:** The updated (2022) CoMiSS (reference #4)

Symptom	Score			
Crying * assessed by parents and without any obvious cause ≥ 1 week	0	≤ 1 h/day		
	1	1–1.5 h/day		
	2	1.5–2 h/day		
	3	2 to 3 h/day		
	4	3 to 4 h/day		
	5	4 to 5 h/day		
	6	≥ 5 h/day		
Regurgitation * ≥ 1 week	0	0–2 episodes/day		
	1	≥ 3– ≤ 5 × of volume < 5 mL		
	2	> 5 episodes of > 5 mL		
	3	> 5 episodes of ± half of the feed in < half of the feeds		
	4	Continuous regurgitations of small volumes > 30 min after each feed		
	5	Regurgitation of half to complete volume of a feed in at least half of the feeds		
	6	Regurgitation of the complete feed after each feeding		
Stools * Brussels Infant and Toddlers Stool Scale (BITSS)	4	Hard stools		
	0	Formed stools		
	4	Loose stools		
	6	Watery stools		
No change ≥ 1 week				
Skin symptoms	0 to 6	Atopic eczema ≥ 1 week		
			Head neck trunk	Arms hands legs feet
		Absent	0	0
		Mild	1	1
		Moderate	2	2
		Severe	3	3
	0 or 6	Acute urticaria * and/or angioedema * (no 0/yes 6)		
Respiratory symptoms * ≥ 1 week	0	No respiratory symptoms		
	1	Slight symptoms		
	2	Mild symptoms		
	3	Severe symptoms		
Additional information to consider				
Worsening of existing eczema might be indicative of CMA				

If urticaria/angioedema can be directly related to cow’s milk (e.g., drinking milk in the absence of other food) this is strongly suggestive of CMA

*In the absence of infectious disease

In 2022, after reviewing evidence from 25 clinical trials, the CoMiSS was revised and updated [13]. The key changes included lowering the cut-off score from ≥ 12 to ≥ 10 to indicate the likelihood of symptoms being related to cow’s milk, and also replacing the Bristol Stool Scale with the Brussels Infant Toddlers Stool Scale (BITSS), as the latter is more appropriate for non-toilet trained children [14]. The panel of experts agreed that CoMiSS also may be potentially useful for infants between 6 and 12 months [13].

However, there is a lack of data on presumed healthy infants in this age range. The aim of this study was to determine the CoMiSS values in presumed healthy infants who have completed 6 months and are up to 12 months old, hereafter referred to as 6 to 12 months old. The purpose of this determination was to facilitate a better interpretation of the CoMiSS values in patients with symptoms within the age range where the diagnosis of CMA is often established.

## Methods

### Study design and data collection

This was an international multicenter cross-sectional study conducted between September 2022 and April 2023. The study involved Investigators from Belgium, Bulgaria, Czech Republic, Italy, Poland, and Spain. Study subjects were enrolled by healthcare professionals at well-baby clinics during routine follow-up visit or vaccinations. All data were collected prospectively.

### Participants

The study focused on presumed healthy infants between completed 6 and 12 months of age. Exclusion criteria included preterm delivery (less than 37 weeks), acute or chronic disease, and the consumption of a therapeutic formula (including partially hydrolyzed formula), dietary supplements (except vitamins), or medication.

### Data collection

The following information was collected: gestational age, gender, age, type of feed (breast milk or infant formula), and complementary feeding. All of this data was recorded in a standardized Excel file (computer program, version 16.78, Microsoft Corporation, 2022). Subsequently, this collected data was combined and summarized at the end of the study.

### CoMiSS assessment

The CoMiSS questionnaires were filled out by the health care providers, based on interviews conducted with the care providers. The interviews were carried out in the native languages of the participating countries, using questionnaires that are available in those local languages.

### Ethical considerations

After obtaining informed consent from caregivers, the CoMiSS questionnaire was anonymously filled out. This study was approved by the respective ethical committees of participating institutions.

### Statistical analysis

Descriptive statistics were employed, including the 5th, 25th, 75th, and 95th centiles, mean and standard deviation (SD) for normally distributed continuous variables, and median and interquartile ranges (IQR) for non-normally distributed variables. The normality was tested by Kolmogorov–Smirnov test. The global distribution of the CoMiSS for each value was calculated. Differences between groups were analyzed using a χ2-test for nominal variables, which are presented as the count and percentage (*n*%). The Mann–Whitney *U* test or Kruskal–Wallis test was used for continuous variables and results are presented as the median (Q1–Q3). Post hoc testing for skewed variables was done using a Nemenyi test. Regression analysis was performed to evaluate the impact of age, gender, country, and feeding type on CoMiSS values. A *p* value of < 0.05 was considered statistically significant. The analysis was performed using the R software (computer program; Version 3.5.1; http://cran.r-project.org).

## Results

### Population description

A total of 609 participants were recruited for the study, with the following distribution across countries: Belgium, 21 (3.5%); Bulgaria, 59 (9.7%); Czech Republic, 55 (9%); Italy, 94 (15%); Poland, 282 (46%); and Spain, 98 (16%). Overall, 333 (54%) boys and 276 (45%) girls were included. In terms of type of feeding type, 210 (34%) were breastfed, 264 (43%) were formula fed, and 135 (22%) were mixed fed. For the purposes of some of the analyses, mixed feeding was combined with formula feeding into one group (*n* = 399). Complementary feeding was introduced in 584 infants (96%). The overall median (Q1–Q3) age was 37 (30–44) weeks. There was no missing data. All study sites reported completed data for infants who met the inclusion criteria. Subjects’ characteristics are presented in Table S1.

### Overall results of the CoMiSS

Descriptive statistics of the CoMiSS in infants aged 6–12 months are presented in Table 2. The overall median (Q1–Q3) CoMiSS values were 3 (1–5). CoMiSS values ≥ 10 were observed in 1.3% of infants (*n* = 8). There were no significant differences in total CoMiSS values according to gender (*p* = 0.55) or type of feed (*p* = 0.9). However, the post hoc analysis showed significantly higher total CoMiSS scores in infants aged 6 months compared to 10 months (*p* = 0.001) and 11 months (*p* = 0.007). The highest median CoMiSS value was observed in infants at 6 months [4], while the lowest median value was observed in infants at 12 months [1].

**Table 2:** Descriptive statistics of the CoMiSS in healthy infants aged 6–12 months

	**P5**	**P25**	**Median**	**P75**	**P95**	**Min–max**
Total (*n* = 609)	0	1	3	5	8	0–12
Crying	0	0	0	1	3	0–6
Regurgitation	0	0	0	1	2	0–5
Stools	0	0	0	4	4	0–6
Skin symptoms (total)	0	0	0	0	1	0–6
Respiratory symptoms	0	0	0	0	1	0–2
Gender						
Boys (*n* = 333)	0	1	2	5	7.4	0–12
Girls (*n* = 276)	0	1	3	5	8	0–12
Feeding type						
Breastfeeding (*n* = 210)	0	1	3	5	7	0–11
Formula feeding (*n* = 264)	0	1	3	5	8	0–12
Mixed feeding (*n* = 135)	0	1	3	4	7.3	0–11
Country						
Belgium (*n* = 21)	0	0	1	4	6	0–7
Bulgaria (*n* = 59)	0	0.5	4	6	8.2	0–10
Czech Republic (*n* = 55)	0	0	1	3.5	5	0–6
Italy (*n* = 94)	0	1	4	5	8.35	0–12
Poland (*n* = 282)	0	1	3	4.75	6	0–9
Spain (*n* = 98)	0	0	3	6	9	0–12
Age						
6 months (*n* = 137)	0	1	4	6	9	0–12
7 months (*n* = 105)	0	1	3	5	8	0–10
8 months (*n* = 104)	0	1	3.5	5	6.85	0–10
9 months (*n* = 84)	0	1	3	5	8.7	0–12
10 months (*n* = 78)	0	0.25	2	4	6	0–8
11 months (*n* = 80)	0	0	2	4	7	0–9
12 months (*n* = 21)	0	0	1	4	7	0–9

The median and interquartile range were consistent among genders and types of feed, except for mixed feeding, where Q3 was 4. The 95th percentile for total CoMiSS was 8 and was consistent with the group of girls and formula-fed infants. The 95th percentile was lower in the group of boys (7.4), breastfed infants [7], and mixed-fed infants (7.3). The distribution of the total CoMiSS values is presented in Fig. S1, Supplemental Materials. In the regression analysis, it was observed that age significantly predicted CoMiSS values, irrespective of the country and feeding method (*β* =  −0.270, *p* < 0.001). Moreover, even when the impact of the feeding method was excluded from the analysis, age continued to be a significant predictor for CoMiSS (*β* =  −0.259, *p* < 0.001).

### Contribution of symptoms to the CoMiSS

The distribution of each symptom in the CoMiSS according to gender, type of feed, and age is presented in Figs. S2, S3, and S4 (supplementary materials), respectively.

#### Crying

There were no differences in the crying score across the gender (p = 0.14), age (*p* = 0.88), or type of feed (*p* = 0.12).

#### Regurgitation

No differences in regurgitation score values were found according to gender (*p* = 0.12) or type of feed (*p* = 0.48). However, there were significant differences observed across age groups (*p* = 0.02). Out of the total participants, 17 infants (2.8%) had a regurgitation score greater than 3. The majority of these infants (10 out of 17) were 6 months old. None of the infants presented with regurgitations of the total volume after each meal (score of 6), and only one infant (0.2%) had regurgitations of more than half of the reported volume in at least half of meals (score of 5).

#### Stools

There were no significant differences in the stools score based on gender (*p* = 0.97) or type of feed (*p* = 0.56). However, there was a significant change in the stools score with age (*p* < 0.001). Significantly higher values were present in infants at 6 months compared to infants at 10 (*p* = 0.01) and 11 months (*p* = 0.02) of age. Among 230 infants with stools score of 4 or above, 70 infants (30%) were aged 6 months.

#### Skin symptoms

No significant differences were found in the total skin score according to age (*p* = 0.69), gender (*p* = 0.37), or feeding type (*p* = 0.15). Urticaria was reported in only one of the included participants.

##### Respiratory symptoms

There were no differences in respiratory symptoms according to gender (*p* = 0.18), age (*p* = 0.13), or feeding type (*p* = 0.72). No infants were reported as having severe respiratory symptoms.

## Discussion

The median CoMiSS value in presumed healthy infants between 6 and 12 months in different European countries was found to be 3, which corresponds with data from infants up to the age of 6 months [15]. While previous data showed a tendency for higher CoMiSS values with increasing age in infants up to 6 months, this study showed a significant decrease in total CoMiSS values in subsequent months, starting from a median of 3 at 6 months and decreasing to a median of 1 at 12 months. A similar trend was observed for the 95th centile. Overall, the 95th centile was 8, with a tendency to be lower in subsequent months, reaching 7 at 12 months. Interestingly, the lowest 95th centile was observed at 10 months and was 6. However, there were relatively few participants in this age group (*n* = 78), which could have influenced the results. Our results emphasize the importance of age in understanding and interpreting CoMiSS values in infants aged 6–12 months. Further research could explore the underlying mechanisms behind this association and its implications for assessing cow’s milk-related symptoms in this age group.

This study has limitations. The study was conducted using a convenience sample of infants attending well-clinics for routine check-ups, rather than employing a random sampling method. This introduces a potential bias, as the characteristics of these infants may differ from those who were not approached or did not participate, thus, limiting the generalizability of the findings to the wider population of healthy infants. The questionnaires used in this study were translated into the native languages of participating countries; however, they were not validated. Although the researchers completed the questionnaire based on the answers in the native language and ensured that it corresponded to the original language of the questionnaire, there is a risk of translation bias. Furthermore, there is a risk of non-response bias, as the participation of parents in the study may have been influenced by factors related to their infant’s health or symptoms. This introduced the possibility of selection bias and limits the representativeness of the included participants. Additionally, there was a discrepancy in the size of cohorts from different countries included in this study. Participants from Poland accounted for close to 47% of all infants analyzed, which could have influenced the results and introduced a potential bias. It is important to consider the impact of this uneven distribution when interpreting the findings.

As stated in the Introduction, CoMiSS was developed as an awareness tool to help healthcare providers identify potential cow’s milk-related symptoms in infants. However, it is important to understand that it is not a diagnostic tool. A definitive diagnosis of CMA requires further tests, such as an oral food challenge. Therefore, CoMISS should only be used as a preliminary step to identify the likelihood of CMA and guide initial management; it should not replace formal diagnostic procedures. Furthermore, although modified CoMiSS scores have been used for predicting lactose intolerance in adults [16] and efforts are made to investigate the relationship between fecal calprotectin levels and CoMiSS scores [17], these applications have not been validated. Therefore, we do not recommend using CoMiSS for purposes other than those for which it was developed.

In conclusion, this study provides CoMiSS values for presumed healthy European infants aged 6–12 months, extending our understanding beyond the commonly studied age range (0–6 months). The influence of age on CoMiSS values highlights the importance of age-specific reference ranges when interpreting CoMiSS values in infants aged 6–12 months. Further research is needed to assess the use of CoMiSS as an awareness tool for cow’s milk-related symptoms for symptomatic infants older than 6 months.

### Supplementary Information

Below is the link to the electronic supplementary material.

Supplementary file1 (DOCX 17 KB)

Supplementary file2 (DOCX 19 KB)

Supplementary file3 (DOCX 19 KB)

Supplementary file4 (DOCX 20 KB)

Supplementary file5 (DOCX 14 KB)

Supplementary file6 (DOCX 15 KB)
