# Supplementary material for: Cow’s Milk-related Symptom Score (CoMiSS) values in presumed healthy European infants aged 6–12 months: a cross-sectional study
Source: Eur J Pediatr. 2023 Nov 17;183(2):707–13. doi: 10.1007/s00431-023-05334-0 (PMC10912251; doi:10.1007/s00431-023-05334-0)
Supplement: Supplementary file 4 — Supplementary file4 (DOCX 20 KB) [file 431_2023_5334_MOESM4_ESM.docx]

**Figure S4. Distribution of CoMiSS according to age.**
