# Supplementary material for: Cow’s Milk-related Symptom Score (CoMiSS) values in presumed healthy European infants aged 6–12 months: a cross-sectional study
Source: Eur J Pediatr. 2023 Nov 17;183(2):707–13. doi: 10.1007/s00431-023-05334-0 (PMC10912251; doi:10.1007/s00431-023-05334-0)
Supplement: Supplementary file 5 — Supplementary file5 (DOCX 14 KB) [file 431_2023_5334_MOESM5_ESM.docx]

**Table S1. Patients’ characteristics**

|  | **n** | **%** |
| --- | --- | --- |
| Total | 609 |  |
| Belgium | 21 | 3.4 |
| Bulgaria | 59 | 9.7 |
| Czech Republic | 55 | 9.0 |
| Italy | 94 | 15.4 |
| Poland | 282 | 46.3 |
| Spain | 98 | 16.1 |
| Boys | 333 | 54.7 |
| Girls | 276 | 45.3 |
| Exclusively breastfed | 210 | 34.5 |
| Non-exclusively breast fed | 399 | 65.5 |
| Median age in weeks | 37 |  |
| (Q1:Q3) | (30;44) |  |
| 6 months | 137 | 22.5 |
| 7 months | 105 | 17.2 |
| 8 months | 104 | 17.1 |
| 9 months | 84 | 13.8 |
| 10 months | 78 | 12.8 |
| 11 months | 80 | 13.1 |
| 12 months | 21 | 3.4 |
