# Supplementary material for: Cow’s Milk-related Symptom Score (CoMiSS) values in presumed healthy European infants aged 6–12 months: a cross-sectional study
Source: Eur J Pediatr. 2023 Nov 17;183(2):707–13. doi: 10.1007/s00431-023-05334-0 (PMC10912251; doi:10.1007/s00431-023-05334-0)
Supplement: Supplementary file 6 — Supplementary file6 (DOCX 15 KB) [file 431_2023_5334_MOESM6_ESM.docx]

**Table S2. Patients’ characteristics by country**

|  | **Belgium**  **N (%)** | **Bulgaria**  **N (%)** | **Czech Republic**  **N (%)** | **Italy**  **N (%)** | **Poland**  **N (%)** | **Spain**  **N (%)** |
| --- | --- | --- | --- | --- | --- | --- |
| Total (n = 609) | 21 (3) | 59 (10) | 55 (9) | 94 (15) | 282 (47) | 98 (16) |
| Boys (n = 333) | 11 | 31 | 29 | 54 | 161 | 47 |
| Girls (n = 276) | 10 | 28 | 26 | 40 | 121 | 51 |
| Breastfed (n = 210) | 4 | 14 | 28 | 32 | 100 | 32 |
| Non-breast fed (n = 399) | 17 | 45 | 27 | 62 | 182 | 66 |
| Median age in weeks | 35 | 35 | 35 | 36 | 38 | Not informed |
| (Q1:Q3) | (30:40) | (28:42) | (27:44) | (28:43) | (33:45) | Not informed |
| 6 months (n = 137) | 5 | 20 | 19 | 27 | 47 | 19 |
| 7 months (n = 105) | 4 | 8 | 6 | 16 | 51 | 20 |
| 8 months (n = 104) | 4 | 13 | 10 | 13 | 55 | 9 |
| 9 months (n = 84) | 4 | 4 | 1 | 16 | 43 | 16 |
| 10 months (n = 78) | 2 | 5 | 17 | 4 | 47 | 3 |
| 11 months (n = 80) | 2 | 9 | 2 | 14 | 34 | 19 |
| 12 months (n =21) | 0 | 0 | 0 | 4 | 5 | 12 |
